# Use of the concordance index for predictors of censored survival data

**DOI:** 10.1177/0962280216680245

**Published:** 2016-12-29

**Authors:** Adam R Brentnall, Jack Cuzick

**Affiliations:** Centre for Cancer Prevention, Wolfson Institute of Preventive Medicine, Queen Mary University of London, UK

**Keywords:** Biomarkers, C-index, discrimination, Pareto model, proportional-hazards model, survival analysis

## Abstract

The concordance index is often used to measure how well a biomarker predicts the time to an event. Estimators of the concordance index for predictors of right-censored data are reviewed, including those based on censored pairs, inverse probability weighting and a proportional-hazards model. Predictive and prognostic biomarkers often lose strength with time, and in this case the aforementioned statistics depend on the length of follow up. A semi-parametric estimator of the concordance index is developed that accommodates converging hazards through a single parameter in a Pareto model. Concordance index estimators are assessed through simulations, which demonstrate substantial bias of classical censored-pairs and proportional-hazards model estimators. Prognostic biomarkers in a cohort of women diagnosed with breast cancer are evaluated using new and classical estimators of the concordance index.

## 1 Introduction

After determining if predictors of censored survival data are significant, a common objective is to measure their predictive strength on a scale that is not sample dependent. A plethora of statistics have been suggested. Some have attempted to transfer the concept of *R*^2^ from linear regression to censored data.^1,2^ In this article we consider use of the concordance index for censored data.

The first part of the paper reviews the concordance index for predictors of censored survival data. The second part develops concordance index estimators that are valid when the strength of the predictor becomes diminished with follow up. Our proposals are compared with classical methods using computer simulations and a breast cancer prognostic biomarker example.

## 2 Concordance index

The concordance index was initially developed to estimate the degree to which a randomly chosen observation from one distribution was larger than one chosen independently from another distribution.^3^ When *T*_1_ and *T*_2_ are continuous independent random variables with cumulative distribution functions *F*_1_ and *F*_2_ the concordance index is

$$\begin{array}{c} C=P(T_{1}>T_{2})\\ =\int \{1-F_{1}(u)\}\text{d}F_{2}(u) \end{array}$$

If *T*_1_ and *T*_2_ place positive mass at the same point then we count half for ties and define *C* as *P*(*T*_1_* > T*_2_) + *P*(*T*_1_ = *T*_2_)/2 so that

(1) $$\begin{array}{c} C=\int \{1-F_{1}(u)+\frac{1}{2}P(T_{1}=u)\}\text{d}F_{2}(u) \end{array}$$

and *C* = 0.5 when the two distributions are the same, even with ties. The concordance index can be estimated from the normalized Wilcoxon ranksum (Mann–Whitney) statistic, by

$$\begin{array}{c} \hat{C}=(\mathrm{nm}{)}^{-1}\sum_{i=1}^{n}\sum_{j=1}^{m}I(T_{1i}>T_{2j})+\frac{1}{2}I(T_{1i}=T_{2j}) \end{array}$$

where *T*_1_*_i_* (*i* = 1, *…*, *n*) and *T*_2_*_j_* (*j* = 1, *…*, *m*) are independent samples from *F*_1_ and *F*_2_ respectively, and *I*(.) denotes the indicator function. If *R_i_* denotes the rank of the *T*_1_*_i_* (*i* = 1, *…*, *n*) in the combined sample (*T*_11_, *…*, *T*_1_*_n_*, *T*_21_, *…*, *T*_2_*_m_*) with the ranks of tied observations averaged, then the Wilcoxon ranksum test statistic is given by $W=\sum_{i=1}^{n}R_{i}$, which can be related to $\hat{C}$ through $W=\mathrm{nm}\hat{C}+n(n+1)/2$. When the samples (*T*_1_ and *T*_2_) come from cases and controls respectively, the concordance index is the area under the receiver operating characteristic curve for (*F*_1_, *F*_2_).^4^ When the samples are from two arms of a randomised control trial, *C* is a measure of the treatment effect. Some variations of *C* have also been studied. These include the odds of concordance *C*(1−*C*)^−1^,^5–7^ and a modification to account for matched case-control designs,^8^ but they are not considered further in this article.

For a one-parameter family {*T_Z_*} of random variables indexed by real number *Z* from distribution {*F_Z_*}, a concordance index that quantifies the degree of association between *T_Z_* and *Z* is defined as

(2) $$\begin{array}{c} {\text{C}}_{Z}=2\int_{z_{1}>z_{2}}\int \{P(T_{z_{1}}>T_{z_{2}})+\frac{1}{2}P(T_{z_{1}}=T_{z_{2}})\}\text{d}F_{Z}(z_{1})\text{d}F_{Z}(z_{2})+\frac{1}{2}P(Z_{1}=Z_{2}) \end{array}$$

where the last term essentially derives from allowing ties in *Z* to be broken at random.^9^ The definition has the advantage of being continuous in the distribution of *F_Z_* and is equivalent to Kendall’s *τ* rank correlation coefficient because $C_{Z}=0.5+\tau /2$.

*C_Z_* and *C* are not the same when *Z* is a two-point distribution, but they are linearly related. Consider where *Z* = 1, 2 (e.g. respectively cases and controls, or treated and untreated) and *P*(*Z* = 1) = *P*(*Z* = 2) = 0.5. Then $C_{Z}=2\times P(T_{2}>T_{1})\times 0.5\times 0.5+1/2\times 0.5=C/2+1/4$. Thus for the balanced two-sample situation the range of *C_Z_* is only (1/4, 3/4) and not (0, 1) as for *C*. This important aspect is due to ties in *Z*, and interpretation of *C_Z_* is affected whenever ties in *Z* are possible. For example, the upper bound of *C_Z_* may decrease if a continuous *Z* is rounded. Although obvious from (2), this might seem surprising because in practice it is often implicitly assumed that the range of the concordance index *C_Z_* is always (0, 1). Some bounds on the range of *C_Z_* are as follows. Suppose there are *n* discrete values of *Z*. Then the smallest possible *P*(*Z*_1_ = *Z*_2_) occurs when they are distributed uniformly so that $P(Z_{1}=Z_{2})=1/n$; the smallest minimum value of *C_Z_* with *n* points is (2*n*)^−1^ and the maximum is $1-(2n{)}^{-1}$. Therefore, with discrete data one might normalize *C_Z_* so that it can theoretically attain 0 and 1 via $\{C_{Z}-(2n{)}^{-1}\}(1-1/n{)}^{-1}$. For large *n* the range of *C_Z_* is less of an issue, and for continuous distributions of *Z* the range of *C_Z_* is (0, 1), as can be seen by letting *T_Z_* = {−*Z*} and *T_Z_* = {*Z*} respectively be a set of degenerate one-point distributions for continuous *Z*.

In the rest of the paper we focus on estimators of *C* and *C_Z_* for right-censored data.

## 3 Estimator review

### 3.1 Censored-pairs estimators

The concordance indices (1) and (2) have been extended to censored data by ignoring pairs when the smaller survival time is censored and using a normalising constant to account for these uninformative pairs.^10,11^ While such statistics can be useful for comparing different models on the same data set, Efron^12^ noted that Gehan’s approach^10^ was dependent on the censoring distribution, and so was not not a universal measure of *P*(*T*_1_* > T*_2_). Others have noted that Harrell’s approach^11^ likewise depends on the censoring distribution.^13^ If the censoring random variable *H_Z_* is conditionally independent of *T_Z_* given *Z*, so that the observed survival function is (1−*F_T_Z__*)(1−*F_H_Z__*), then from equation (2), the censored-pairs concordance index is given by

(3) $$\begin{array}{c} C_{\mathrm{ZH}}=[2\int_{z_{1}>z_{2}}\int \{P(T_{z_{1}}>T_{z_{2}})+\frac{1}{2}P(T_{z_{1}}=T_{z_{2}})\}\\ \times P(H_{z_{1}}>T_{z_{2}})P(H_{z_{2}}>T_{z_{2}})\text{d}F_{Z}(z_{1})\text{d}F_{Z}(z_{2})+\frac{1}{2}P(Z_{1}=Z_{2})]\\ \times [2\int_{z_{1}>z_{2}}\int P(H_{z_{1}}>T_{z_{2}})P(H_{z_{2}}>T_{z_{2}})\text{d}F_{Z}(z_{1})\text{d}F_{Z}(z_{2})+\frac{1}{2}P(Z_{1}=Z_{2}){]}^{-1} \end{array}$$

The $P(H_{z_{1}}>T_{z_{2}})P(H_{z_{2}}>T_{z_{2}})$ terms in the numerator and denominator arise because contributions to the statistic only occur for pairs of observations when the smaller survival time is not censored. The following methods were developed to be independent of the censoring distribution.

### 3.2 Efron’s estimator of *C*

For the two-sample situation, Efron^12^ suggested a solution using the Kaplan–Meier estimates for the survival distribution given by *S*_1_(*t*) = 1−*F*_1_(*t*) and *S*_2_(*t*) = 1−*F*_2_(*t*), and computing *P*(*T*_1_* > T*_2_) based on these estimates through

$$\begin{array}{c} {\hat{C}}_{E}=-\int {\hat{S}}_{1}(u)\text{d}{\hat{S}}_{2}(u) \end{array}$$

where ${\hat{S}}_{1}$(*u*) and ${\hat{S}}_{2}$(*u*) are the Kaplan–Meier estimates of the survival functions *S*_1_ and *S*_2_ respectively.^14^ That is

(4) $$\begin{array}{c} {\hat{C}}_{E}=(\mathrm{nm}{)}^{-1}\sum_{i=1}^{n}\sum_{j=1}^{m}\hat{Q}(t_{1i},t_{2j},y_{1i},y_{2j}) \end{array}$$

where the observed data are in pairs of event times and indicators (*t*_1_*_i_*, *y*_1_*_i_*) in group 1 and (*t*_2_*_j_*, *y*_2_*_j_*) in group 2, where *y*_1_*_i_* = 0 if *t*_1_*_i_* is censored, one otherwise, and similarly for *y*_2_*_j_*, and $Q(t_{1i},t_{2j},y_{1i},y_{2j})=P(T_{1}>T_{2}|t_{1i},t_{2j},y_{1i},y_{2j})$ is estimated by substituting Kaplan–Meier estimates of survival functions into the relevant terms in Table 1. Examples to show the difference between $E({\hat{C}}_{E})$ and the censored-pairs approach have been reported.^15^

**Table 1.** Values of Efron’s *Q*(*t_i_*, *t_j_*, *y_i_*, *y_j_*) for the concordance statistic. Note that for the two-sample estimator of *C* the 1 and 2 subscripts have been dropped, so that for example *t_i_* represents *t*_1_*_i_* and *t_j_* is *t*_2_*_j_*, similarly *S_i_* is *S*_1_ etc. This notation is used so that the table generalises to estimators of *C_Z_*.

(*y_i_*, *y_j_*)	*t_i_* ≥ *t_j_*	*t_i_ < t_j_*
(1, 1)	1	0
(0, 1)	1	$\frac{S_{i}(t_{j})}{S_{i}(t_{i})}$
(1, 0)	$1-\frac{S_{j}(t_{i})}{S_{j}(t_{j})}$	0
(0, 0)	$1-\frac{S_{j}(t_{i})}{S_{j}(t_{j})}+\frac{\int_{t_{i}}^{\infty }S_{i}(u)\text{d}F_{j}(u)}{S_{i}(t_{i})S_{j}(t_{i})}$	$\frac{\int_{t_{j}}^{\infty }S_{i}(u)\text{d}F_{j}(u)}{S_{i}(t_{i})S_{j}(t_{j})}$

${\hat{C}}_{E}$ overcomes limitations of the censored-pairs approach for the two-group problem but requires that the estimated survival functions decrease to zero, so that one treats the last event time in each group as not censored in the Kaplan–Meier estimator. When there is censoring due to incomplete follow up, with everyone censored by *t*_max_ and where *S*_1_(*t*_max_)* > *0 and *S*_2_(*t*_max_)* > *0, then Efron’s estimator may be very unstable. An important example of this situation is when individuals are enrolled sequentially in a clinical trial and events are recorded until (say) 10-years after the first entry (*t*_max_ = 10). In such situations taking the last time in each group to be an event will substantially bias the concordance index in the direction of the group with the longest surviving member beyond that time. For example, if 90% are at risk in both groups after the last event has occurred, then 81% of the terms in the double summation (4) will favour the group with the longest surviving (censored) member, and ${\hat{C}}_{E}$ is guaranteed to be greater than 0.81−0.19 = 0.62.

### 3.3 Uno’s estimator of *C_Z_*

Uno and colleagues^13^ developed a censored-pairs estimator of the concordance index (2) based on inverse probability weighting. Their solution uses a Kaplan–Meier estimate of the censoring distribution *S_H_*, treating it as independent of *Z* and *T_Z_*, and re-weights the censored-pairs contribution when *t_i_ > t_j_* to be ${\hat{S}}_{H}(t_{j}{)}^{-2}$, rather than one. The approach is justified by inspection of (3); the weighting cancels out the $P(H_{z_{1}}>T_{z_{2}})P(H_{z_{2}}>T_{z_{2}})$ terms, so that it is (asymptotically) independent of the censoring distribution and converges to *C_Z_*.

However, the resulting estimator is only completely independent of the censoring distribution if, as above for the Efron estimator, the maximal follow up for all patients is to a time *τ* such that the marginal survival distribution *S*(*τ*) = *P*(*T > τ*) = 0. If not, then the censored-pairs approach will converge to a quantity greater than *C_Z_*. Informally, this is because the individuals with high *Z* have the event first whether or not hazards also converge with time. More formally, this may be seen by re-expressing *C_Z_* as

(5) $$\begin{array}{c} C_{Z}=\int_{0}^{\infty }C_{t}\frac{S(t)}{\int_{0}^{\infty }S(u)\text{d}u}\text{d}t \end{array}$$

where $S(t)=\int P(T>t|z)\text{d}F_{Z}(z)$ and

(6) $$\begin{array}{c} C_{t}=\int \int \{P(z>z^{*})+\frac{1}{2}P(z=z^{*})\}\text{d}F_{Z}(z|T>t)\text{d}F_{Z}(z^{*}|T=t) \end{array}$$

where $\text{d}F_{Z}(z^{*}|T=t)=\lambda (t|z^{*})/\int \lambda (t|u)\text{d}F_{Z}(u|T=t)$ and $\text{d}F_{Z}(z|T>t)=P(T>t|z)\text{d}F_{Z}(z)/S(t)$ from Bayes’ rule. As *t* increases, the distribution of *Z* in those still at risk becomes weighted towards those with longer survival, and *C_t_* decreases. When follow up is until *t* = *τ*, the censored-pairs concordance index converges to

$$\begin{array}{c} \int_{0}^{\tau }C_{t}\frac{S(t)}{\int_{0}^{\tau }S(u)\text{d}u}\text{d}t \end{array}$$

and because *C_t_* is decreasing this limit is greater than *C_Z_* (anti-conservatively biased) unless *S*(*τ*) = 0. One can also see that the limit of Uno’s concordance index for *τ* close to the longest follow up will be less than Harrell’s version, since it gives relatively more weight to those *C_t_* that are closer to *t* = *τ*.

### 3.4 Proportional-hazards model

A common approach is to estimate linear predictors of outcomes with censored event times using a proportional-hazards model. Here an estimator of the concordance index that does not depend on the censoring distribution or follow up was achieved by Gönen and Heller.^16^ If *T_Z_* has hazard of form $\lambda (T|Z)=\lambda_{0}(T)g(Z)$, then, because

(7) $$\begin{array}{c} P(T_{Z_{1}}>T_{Z_{2}})=\frac{g(Z_{2})}{g(Z_{1})+g(Z_{2})} \end{array}$$

we have from (2) that

(8) $$\begin{array}{c} C_{Z}=2\int_{z_{1}>z_{2}}\int \frac{g(z_{2})}{g(z_{1})+g(z_{2})}\text{d}F_{Z}(z_{1})\text{d}F_{Z}(z_{2})+\frac{1}{2}P(Z_{1}=Z_{2}) \end{array}$$

where *Z*_1_ and *Z*_2_ are independent samples from distribution function *F_Z_*. When $z=\beta_{1}x_{1}+\ldots +\beta_{k}x_{k}$ for some linear combination of covariates ***x*** = (*x*_1_, *…*, *x_k_*) and coefficients $\beta =(\beta_{1},\ldots ,\beta_{k})$, g(.)=exp(.) and both *T_Z_* and *Z* are continuous, the concordance index depends on the distribution of *z* and equals

(9) $$\begin{array}{c} C_{Z}=2\int_{z_{1}>z_{2}}\int \frac{1}{1+\text{exp}(z_{1}-z_{2})}\text{d}F_{Z}(z_{1})\text{d}F_{Z}(z_{2})=2\text{E}[I(Z_{1}>Z_{2})\{1+\text{exp}(Z_{1}-Z_{2}){\}}^{-1}] \end{array}$$

which is linked to *T_Z_* only through the distribution of the coefficients ***β*** and covariates ***x***. Equation (9) may be estimated by replacing *F_Z_* with its empirical distribution so that

(10) $$\begin{array}{c} {\hat{C}}_{Z}=2\{N(N-1){\}}^{-1}\sum_{i=1}^{N-1}\sum_{j=i}^{N}\frac{I({\hat{z}}_{i}>{\hat{z}}_{j})}{1+\text{exp}({\hat{z}}_{i}-{\hat{z}}_{j})} \end{array}$$

where ${\hat{z}}_{i}$ uses the proportional-hazards estimates ${\hat{\beta }}_{1},\ldots ,{\hat{\beta }}_{k}$, and similarly for the more general (8). Its variance is estimable from re-sampling methods or from asymptotic formulae^16^ which depend on the covariance matrix of ***β*** that is routinely available from the partial-likelihood methods of the proportional-hazards model.

## 4 New estimators

### 4.1 Motivation

The methods reviewed above are not universal when the predictor loses strength with time, and may depend on the length of follow up. In particular, formulas (8) and (9) depend implicitly on the validity of the proportional-hazard assumption. Further developments would be useful because hazards are often observed to converge, so that the effect of a predictive factor diminishes as follow-up time increases. This issue is pervasive in applications^5^. For example, in breast cancer epidemiology, many prognostic factors are based on characteristics of the tumour that lose relevance once an individual has survived a period of time^17^. We next propose modifications to the Efron and the proportional-hazard estimators, before introducing a more parsimonious approach.

### 4.2 Modified two-sample estimator

Recall that when there is censoring due to incomplete follow up, Efron’s estimator may be very unstable. The following modification of Table 1 solves this problem by accounting for when the last time in each group is censored.

Denote $A_{i}=(t_{1i}<T_{1}\leq t_{\max})$, $B_{j}=(t_{2j}<T_{2}\leq t_{\max})$, $a=(T_{1}>t_{\max})$ and $b=(T_{2}>t_{\max})$. Let $w_{1i}=P(a|T_{1}>t_{1i})=S_{1}(t_{\max})/S_{1}(t_{1i})$ and $w_{2j}=S_{2}(t_{\max})/S_{2}(t_{2j})$, being respectively defined to be zero when $S_{1}(t_{\max})=0$ or $S_{2}(t_{\max})=0$. Now when $y_{1i}=y_{2j}=0$, *P*(*T*_1_* > T*_2_) may be partitioned as

$$\begin{array}{c} P(T_{1}>T_{2}|A_{i},B_{j})P(A_{i},B_{j})+P(T_{1}>T_{2}|a,B_{j})P(a,B_{j})+P(T_{1}>T_{2}|a,b)P(a,b) \end{array}$$

since $P(T_{1}>T_{2}|A_{i},b)=0$. Then $Q(t_{1i},t_{2j},y_{1i}=0,y_{2j}=0)$ from Table 1 is redefined to be *t*_1_*_i_* ≥ *t*_2_*_j_*

$$\begin{array}{c} \{1-\frac{S_{2}(t_{1i})}{S_{2}(t_{2j})}+\frac{\int_{t_{1i}}^{t_{\max}}S_{1}(u)\text{d}F_{2}(u)}{S_{1}(t_{1i})S_{2}(t_{2j})}\}(1-w_{1i})(1-w_{2j})+w_{1i}(1-w_{2j})+\frac{w_{1i}w_{2j}}{2} \end{array}$$

*t*_1_*_i_ < t*_2_*_j_*

$$\begin{array}{c} \{\frac{\int_{t_{2j}}^{t_{\max}}S_{1}(u)\text{d}F_{2}(u)}{S_{1}(t_{1i})S_{1}(t_{2j})}\}(1-w_{1i})(1-w_{2j})+w_{1i}(1-w_{2j})+\frac{w_{1i}w_{2j}}{2} \end{array}$$

The terms are estimated by using Kaplan–Meier estimates of *S*_2_(*t*) for *w*_2_*_j_*; for example *S*_1_(*t*_max_) is the Kaplan–Meier estimate at the last non-censored time in the first group.

As the original Efron estimator, the modified estimator is not a universal measure when censoring is due to incomplete follow up because it depends on *t*_max_, but it is more stable than the Efron estimator because it does not depend on which group has the longest surviving censored member. It is not consistent for the concordance index if $S_{1}(t_{\max})>0$ and $S_{2}(t_{\max})>0$ but, in this case, clearly it is not possible to obtain a consistent estimator of the concordance index with making assumptions. However, one may obtain an estimate of the concordance index for different follow-up periods by varying *t*_max_, where the modified estimator consistently estimates

$$C_{E}(t_{\max})=-\int_{0}^{t_{\max}}S_{1}(u)\text{d}S_{2}(u)$$

Thus, one approach to facilitate comparisons between studies is to present the estimate of this for different values of *t*_max_. This idea has been used in a similar context elsewhere,^6,13^ and is considered further in later simulations (Figure 2) and an example (Figure 5).

**Figure 1.**
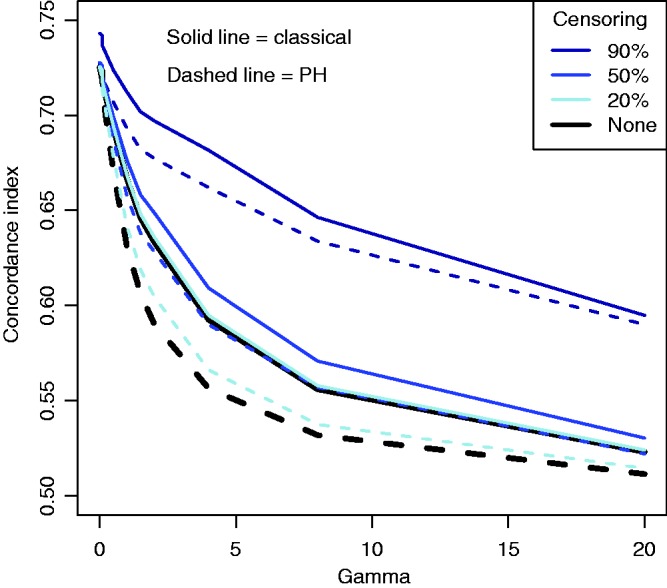
Illustration of the effect of converging hazards and censoring on concordance index estimators. Solid lines (—) use the classical censored-pairs approach, and the proportional-hazards model estimator is dashed (– – –). The true concordance index for this model is when there was no censoring (— black).

**Figure 2.**
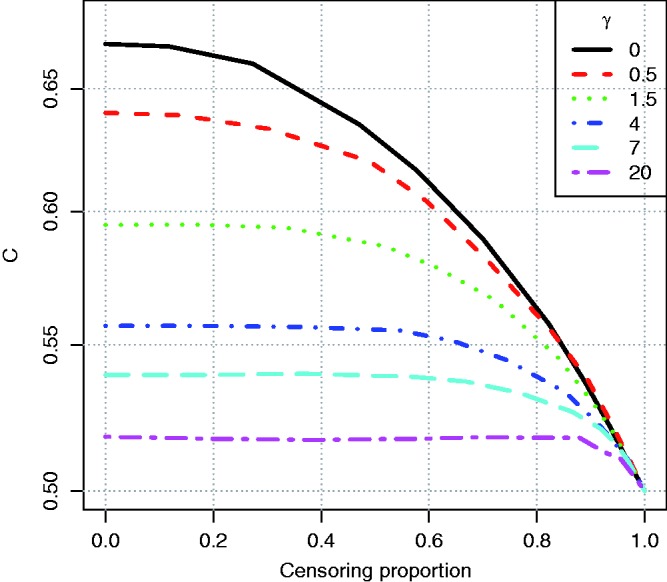
Illustration of the effect of censoring on the two-group concordance statistic estimator. The lines show the concordance index under a Pareto model, with the *γ* parameter shown in the key.

**Figure 3.**
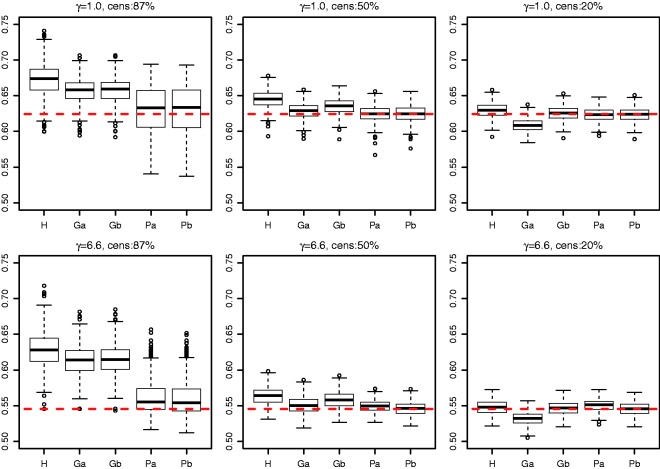
Concordance index estimates from simulations and true value (– – –). H: censored-pairs estimator; Ga: proportional-hazards estimator (10); Gb: hybrid proportional-hazards estimator based on (11); Pa: Pareto estimator using model fit; Pb: hybrid Pareto estimator using Table 1.

**Figure 4.**
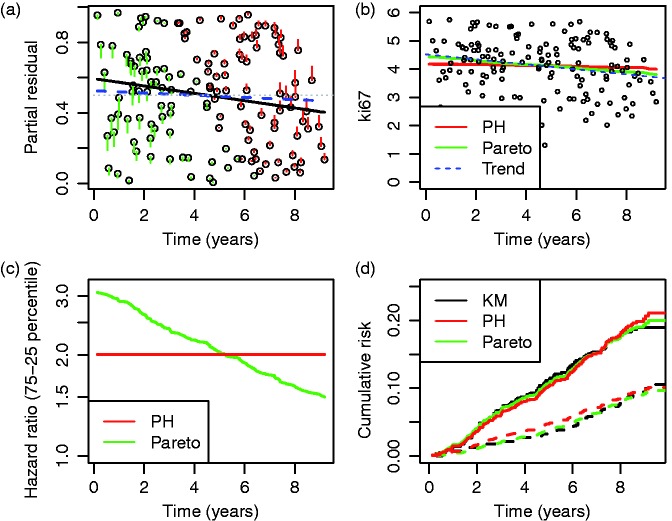
Pareto model fit in example. Plot (a) is Schoenfeld partial residuals from a proportional-hazards (o) and Pareto model (end of line linked to o). Least squares trend lines of the residuals are shown for the proportional-hazards (—) and Pareto models (– –); the line at 0.5 indicates good model fit (- - -). Plot (b) compares the expected Ki67 at each event from the two models and least squares trend line. Plot (c) shows the fitted hazard ratios. Plot (d) is the estimated cumulative risk for a binarised Ki67 in the data (KM, Kaplan–Meier) and the models (— above median, – – – less than or equal to median).

**Figure 5.**
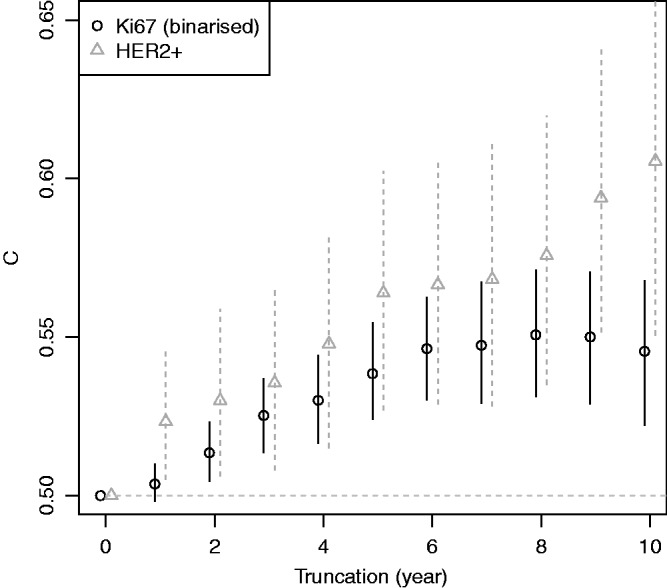
Plot of two-sample concordance index against type I censoring time (*t*_max_) for binarized Ki67 and HER2 from the example. Point-wise 95% confidence intervals (empirical bootstrap) are also shown.

### 4.3 Modified proportional-hazards model estimator

A problem with the estimator of Gönen and Heller^16^ is that if there is no censoring but proportional hazards do not hold, then the estimator will not agree with the classical approach. A partial solution to this is to modify the approach of Efron and write

(11) $$\begin{array}{c} C_{\mathrm{EZ}}=2\{N(N-1){\}}^{-1}\sum_{i=1}^{N-1}\sum_{j=i}^{N}Q(t_{i},t_{j},y_{i},y_{j},z_{i},z_{j}) \end{array}$$

where $Q(t_{i},t_{j},y_{i},y_{j},z_{i},z_{j})=P\{T_{i}>T_{j}|(t_{i},y_{i}),(t_{j},y_{j}),z_{i},z_{j}\}$. Under a proportional-hazards model, *C_EZ_* may be estimated via the terms in Table 1, but the proportional-hazard assumption is only needed to calculate the non-trivial terms and so the estimator agrees with the classical formula when there is no censoring. A further difference to the above is that it requires an estimate of the baseline survivor function *S*_0_(*t*). This approach will be anti-conservatively biased when the data are censored and proportional hazards hold. It is intended for use when censoring is light and one would like robustness against large departures from proportional hazards.

One might consider allowing $\lambda (T|Z)=\lambda_{0}(T)g_{T}(Z)$ for time-varying hazards *g_T_*. In this case

(12) $$\begin{array}{c} P(T_{z_{1}}>T_{z_{2}})=\int_{0}^{\infty }\lambda_{0}(t)g_{t}(z_{2})\text{exp}[-\int_{0}^{t}\lambda_{0}(s)\{g_{t}(z_{1})+g_{t}(z_{2})\}\text{d}s]\text{d}t \end{array}$$

A concordance index based on this involves *O*(*N*^2^) evaluations of this double integral, which would need to be evaluated numerically. One also cannot use the model beyond the maximal follow-up time.

### 4.4 Pareto model

A parsimonious approach is to use a simple one-parameter model to account for varying degrees of convergence by introducing an unobserved additive covariate (frailty) to the proportional-hazards model, independent from other covariates, with a log-gamma distribution with mean one and variance *γ*.^18^ This leads to a transformation model based on the Pareto distribution, so that if the baseline hazard and cumulative hazard are given by *λ*_0_(*t*) and Λ_0_(*t*) respectively, then an individual with covariate z=exp(βx') has survival function

(13) $$\begin{array}{c} S(t|z;\gamma )=1-F_{z,\gamma }(t)\\ =\{1+\gamma z\Lambda_{0}(t){\}}^{-1/\gamma } \end{array}$$

and hazard function

(14) $$\begin{array}{c} \lambda (t|z;\gamma )=z\lambda_{0}(t)\{1+\gamma z\Lambda_{0}(t){\}}^{-1} \end{array}$$

This very flexible model has some attractive features. The hazard ratio is given by

$$\begin{array}{c} \frac{\lambda (t|z_{1};\gamma )}{\lambda (t|z_{2};\gamma )}=\frac{1+\gamma \Lambda_{0}(t)}{z_{2}/z_{1}+\gamma \Lambda_{0}(t)} \end{array}$$

so that a consequence of the frailty (*γ > *0) is that the hazard ratio approaches one as *t* gets large. When *γ* = 0 there is no frailty and it becomes the proportional-hazards model; when *γ* = 1 it becomes the proportional-odds model.

Technical aspects of estimation and inference are considered in the appendix.

#### 4.4.1 Concordance index

Computation of the Pareto concordance index involves a formula with *γ*, the {*Z*} and the baseline cumulative hazard function $\Lambda_{0}(t)$

(15) $$\begin{array}{c} P(T_{z_{1}}>T_{z_{2}}|T_{z_{1}},T_{z_{2}}>s)=\int_{s}^{\infty }\{1+\gamma z_{1}\Lambda_{0}(t){\}}^{-1/\gamma }z_{2}\lambda_{0}(t)\{1+\gamma z_{2}\Lambda_{0}(t){\}}^{-(1+1/\gamma )}\text{d}t\\ =\gamma^{-1}\int_{v}^{\infty }\{1+(z_{1}/z_{2})u{\}}^{-1/\gamma }(1+u{)}^{-(1+1/\gamma )}\text{d}u \end{array}$$

where $v=\gamma z_{2}\Lambda_{0}(s)$, and analysis of concordance index (2) can proceed as the two previous approaches for proportional hazards. That is, the Pareto model can be used with $\{1+\text{exp}(Z_{1}-Z_{2}){\}}^{-1}$ in (9) replaced by (15) with *s* = 0 or via the hybrid approach replacing the non-trivial terms in Table 1 with the Pareto terms. The integral in (15) is needed for both approaches. Although it does not appear to be analytically tractable it may be estimated numerically, and it requires much less computation than (12).

#### 4.4.2 Goodness-of-fit

We lastly consider model goodness-of-fit, partly because the Pareto concordance index is not needed when a proportional-hazards assumption is appropriate. One method is an asymptotic score test for when a Pareto model is taken as the alternative hypothesis to proportional hazards.^19^ Another approach in this line is to apply a likelihood-ratio test for *γ* = 0,^20^ with adjustment for model-boundary testing.^21^ Schoenfeld residuals^22^ are sometimes used, and in the general setting are defined for all i=1,…,N when a non-censored event occurred (*y_i_* = 1) to be

$$\begin{array}{c} {\hat{s}}_{i}=x_{i}-\hat{\text{E}}(x|t\geq t_{i}) \end{array}$$

where

$$\begin{array}{c} \hat{\text{E}}(x|t\geq t_{i})=\frac{\sum_{j=1}^{N}I(t_{j}\geq t_{i})\hat{\lambda }(t_{i}|x_{j})x_{j}}{\sum_{j=1}^{N}I(t_{j}\geq t_{i})\hat{\lambda }(t_{i}|x_{j})} \end{array}$$

and $\hat{\lambda }(t_{i}|x_{j})$ are model estimates. These residuals show the difference between the observed and expected covariate at each event time, and have expectation zero if the model is correct. Plots of *ŝ_i_* against *t_i_* and fitted trends may help to identify departures from the model, and a chi-squared test based on scaled residuals is commonly used to test a proportional-hazards assumption,^23^ without taking a Pareto model as the alternative. Because Schoenfeld residuals were designed to check the proportional-hazard assumption, a direct comparison with the Pareto model will help assess whether it satisfactorily addressed lack of fit. A related goodness-of-fit test is to use partial residuals $\hat{P}(x\geq x_{i})$ defined as^22^

(16) $$\begin{array}{c} {\hat{r}}_{i}=\frac{\sum_{j=1}^{N}I(t_{j}\geq t_{i})\hat{\lambda }(t_{i}|x_{j})I(x_{j}>x_{i})}{\sum_{j=1}^{N}I(t_{j}\geq t_{i})\hat{\lambda }(t_{i}|x_{j})} \end{array}$$

Under the model these should be distributed uniformly between zero and one, independently of *t_i_*. Empirical distribution function goodness-of-fit tests^24^ could be used to assess the distribution of *r_i_* in early and late periods.

## 5 Simulations

### 5.1 Bias

A simulation was used to demonstrate issues with existing methodology when there are converging hazards. Twenty-thousand individuals were simulated with survival times from a Pareto distribution; the rate for an individual was the exponent of a random normal covariate with unit mean and variance multiplied by a frailty sampled from a gamma distribution with mean one and variance *γ*. Type I censoring was considered, so that events occurred before a maximal follow-up time based on the expected proportion censored. For exposition we show 90%, 50% and 20% censoring. For ∼10-year follow up, heavy censoring might correspond to survival such as for distant recurrence in women diagnosed with estrogen-receptor positive breast cancer;^25^ mid-range censoring (∼50%) might be seen for survival following disease such as an acute myocardial infarction event;^7^ light censoring occurs when survival rates are low, for example, for survival following complete resection of non-small-cell lung cancer.^5^ In all simulation scenarios there is no difference between the censored-pairs estimators of Harrell or Uno because everyone is censored at the same time. Concordance indices using a proportional-hazards model and the censored-pairs statistic were calculated and compared with the true index, obtained using a simulation without censoring.

The results in Figure 1 show that for this model the proportional-hazard estimate was conservative when there was no censoring, but had positive bias when censoring was more than about 50%. The classical estimator substantially overestimated the concordance index when censoring was 50% or more; this bias was more pronounced for heavy censoring as the frailty variance *γ* increased.

A second simulation was used to demonstrate the dependence of the two-sample estimator on follow up. Ten-thousand individuals were simulated in two groups, with survival time from an exponential distribution with rate one or two, compounded with a gamma frailty with variance *γ*, which was chosen to show the effect of a change from constant hazards (*γ* = 0) to when they converge very quickly (*γ* = 20). Censoring was generated by allowing individuals to be enrolled into a study at different times according to a uniform distribution between [0.00, 0.05], and then they were censored at a maximum follow-up time. The results in Figure 2 show that the two-sample statistic was conservatively biased when there was heavy censoring. Considering the chart from right (heavy censoring due to censoring) to left (no censoring), one can see that the concordance index estimate increased with more follow up (later censoring) until the covariate had ceased to influence survival due to converging hazards. The plot shows that the statistic is actually better when there are converging hazards, since it will converge to the true value with less follow up.

### 5.2 Comparison of estimators

A final simulation was used to compare estimators of *C_Z_*. Survival times were from a Pareto distribution that was the exponent of a standard random normal covariate (*x*) multiplied by 0.7 (i.e. *z* = exp(*βx*) with *β* = 0.7) and compounded by a frailty sampled from a gamma distribution with mean one and variance *γ*. Two choices of *γ* were considered (1.0 and 6.6) and three levels of censoring (follow up to time with expected censoring percentage 87%, 50% and 20%). The sample size was 1125 and 500 replications were used. The Pareto model was fitted by maximizing the profile likelihood (see Appendix).

The reason for choosing *β* = 0.7, *γ* = 6.6, 87% censoring and *n* = 1125 is that these correspond to an example in the next section (Table 3(b), Ki67). We also considered *γ* = 1 in order to assess a scenario where the proportional-hazards assumption is violated more slowly, and partly for theoretical interest because it corresponds to a proportional-odds model. The censoring levels were varied to help assess the estimators as more follow up is accrued.

The distribution of estimated concordance indices is shown in Figure 3. The concordance-index estimates from a Pareto model were substantially less biased than the other methods with heavy censoring (Table 2). The Pareto estimator was biased for heavy censoring at this sample size because it fits a proportional-hazards model where there is insufficient power to detect non-proportional hazards. Harrell’s statistic and the modified proportional-hazards statistic became less biased as the level of censoring decreased. The Pareto estimator had a lower mean squared error than the other estimators (Table 2).

**Table 2.** Simulation estimation results for two scenarios of *γ*.

	Mean bias (×100)			MSE (×100)		
Censoring:	87%	50%	20%	87%	50%	20%
*γ* = 1 (proportional odds)						
Censored pairs	4.8	2.1	0.5	28.4	5.7	1.2
PH-fit	3.2	0.4	−1.6	13.7	1.4	3.3
PH-hybrid	3.3	1.1	0.1	14.1	2.4	0.9
Pareto-fit	0.6	0.0	−0.1	10.5	1.3	0.8
Pareto-hybrid	0.6	0.0	−0.1	10.7	1.4	0.9
*γ* = 6.6						
Censored pairs	8.3	1.8	0.2	75.5	4.8	1.0
PH-fit	6.8	0.5	−1.3	50.3	1.7	2.6
PH-hybrid	6.9	1.2	0.1	51.8	3.0	1.0
Pareto-fit	1.7	0.4	0.5	9.4	0.9	0.9
Pareto-hybrid	1.6	0.1	0.0	9.2	0.9	0.9

MSE: mean squared error; PH-fit: proportional-hazards estimator (10); PH-hybrid: proportional-hazards estimator based on (11); Pareto-fit: estimate using model fit only; Pareto-hybrid: Pareto model estimator using Table 1.

Some differences were seen between a proportional-hazards concordance index based solely on model fit and the hybrid approach using Table 1. As expected the hybrid approach worked best for light censoring. It was worse under 50% censoring for the proportional-hazards model because it shifted the estimate towards the Harrell estimate, and the censored-pairs estimators are expected to be anti-conservative unless follow up is to a point where survival is zero (c.f. Figure 1). Thus, we do not recommend the hybrid approach unless censoring is light.

## 6 Example

The example uses a sample of 1125 women with oestrogen-receptor positive breast cancer, of whom 145 had a distant recurrence after a median 8.5-years follow up in a clinical trial (ATAC trial, ISRCTN registration numer ISRCTN18233230). This sample from the transATAC study (approved by the South-East London Research Ethics Committee (REC ref no. 971037)) were previously used to show that some immunohistochemical (IHC) biomarkers added useful information to classical clinical prognostic factors.^25^ For demonstration and insight we focus next on some of the individual biomarkers used in the IHC risk score. We do not present results from the hybrid estimators because censoring is heavy, but there was little difference because model assumptions dominate the calculations (87% of women were censored).

Table 3 shows some univariate concordance index estimates. The following points are of note. First, the two-sample estimates were different than the other form of concordance index. Second, Harrell’s and Uno’s statistics were closer to each other than the proportional-hazards and Pareto model statistics. This is likely due to the bias from follow up, as discussed earlier. Third, Pareto estimates were substantially lower than the proportional-hazards model when $\hat{\gamma }\neq 0$, reflecting an assumption of converging hazards. Finally, the concordance indices of binarised predictors were less than continuous counterparts due to the information loss from dichotomising.

**Table 3.** Estimated univariate concordance indices and model coefficients from example.

	Grade	HER2	Nodes	Ki67	ER
(a) Binary predictor					
2-sample	0.57	0.61	0.59	0.55	0.53
Harrell	0.59	0.57	0.63	0.61	0.56
Uno	0.58	0.57	0.63	0.58	0.56
PH	0.57	0.54	0.60	0.59	0.56
Pareto	0.53	*	*	0.53*	
PH $\hat{\beta }$(LR-*χ*^2^)	0.9 (24.9)	1.1 (23.1)	1.2 (47.5)	0.8 (21.6)	−0.5 (7.8)
Pareto $\hat{\beta }$(LR-*χ*^2^)	1.3 (27.0)	*	*	1.4 (25.2)*	
$\hat{\gamma }$(LR-*χ*^2^)	4.0 (2.1)	0.0 (0.0)	0.0 (0.0)	8.7 (3.6)	0.0 (0.0)
(b) Continuous predictor					
Harrell			0.65	0.64	0.57
Uno			0.64	0.62	0.58
PH			0.61	0.63	0.57
Pareto			*	0.55	0.54
PH $\hat{\beta }$(LR-*χ*^2^)			1.0 (72.7)	0.4 (31.8)	−0.2 (11.5)
Pareto $\hat{\beta }$(LR-*χ*^2^)			*	0.7 (35.2)	−0.2 (12.0)
$\hat{\gamma }$(LR-*χ*^2^)			0.0 (0.0)	6.6 (3.5)	2.8 (0.4)

PH: using proportional-hazards assumption and (10); Grade: moderate or worse; HER2: positive; Nodes: lymph node positive or number of nodes (ordinal: 0, 1–3,* > *4); Ki67: above median or continuous marker; ER: oestrogen-receptor score above median or continuous; LR-*χ*^2^: likelihood-ratio statistic; $\hat{\beta }$: estimated regression coefficient for predictor; * indicates when Pareto model fit was proportional hazards.

To explore further we focus on Ki67, whose Pareto concordance index estimate was 0.552 (SE (standard error) 0.0156) compared with 0.631 (SE 0.0210) under a proportional-hazards assumption, 0.644 (SE 0.0220) for Harrell’s estimator and 0.624 (SE 0.0213) for Uno’s adjusted version. Ki67 showed evidence of a departure from proportional hazards, seen informally by inspection of Table 4. More formally, a likelihood-ratio test (Table 3) that *γ* = 0 had *P* = 0.03 (after correction for model-boundary testing^21^); a different test for non-proportionality^23^ yielded *χ*_1_^2 ^= 4.16, *P* = 0.04. Schoenfeld partial residuals in Figure 4(a) show that allowing for converging hazards via a Pareto model improved the residuals at the start and end. Figure 4(b) helps to show why; the expected value of Ki67 for events decreased more rapidly than a proportional-hazards assumption. Figure 4(c) shows the fitted hazard ratio from the Pareto model, which approximately halved over the period. Figure 4(d) demonstrates that a Pareto model for a binary Ki67 predictor better matched the Kaplan–Meier estimates than a proportional-hazards model.

**Table 4.** Number of events in each year, split by Ki67 median (low/high).

Year	Low Ki67	High Ki67	Ratio
1	4	8	2.0
2	3	14	4.7
3	3	16	5.3
4	5	10	2.0
5	4	13	3.2
6	4	12	3.0
7	9	9	1.0
8	8	10	1.2
9	6	5	0.8
10	2	0	0.0

A goodness-of-fit test of the Pareto model is suggested by Figure 4(a), where most of the change in partial residuals between the proportional-hazards and Pareto model were in the first and last three years. Applying a two-sample Kolmogorov–Smirnov test of equality in distribution between the residuals in years ≤ 3 vs* > *6 for the proportional-hazards model was rejected (*D* = 0.28, two-sided *P* = 0.03). The trend line shows that the Pareto model fitted somewhat better, and the same test did not reject a fit of the Pareto model (*D* = 0.22, *P* = 0.17). Thus the data showed some evidence to support the Pareto model fit, which was certainly better than proportional hazards, and the lower concordance index estimate than from a proportional-hazards model or the other approaches.

Figure 5 plots the two-sample concordance index for binarised Ki67 by censoring time. The concordance index increased, and then appeared to plateau after six years. Thus one might surmise that the two-sample estimate from 10-year follow up is unlikely to increase for this variable with further follow up due to converging hazards (c.f. Figure 2). HER2 positivity is included for comparison, where the estimated concordance index increased with follow up, in better agreement with a proportional-hazards assumption.

## 7 Conclusion

The concordance index is routinely used to measure how well a variable predicts the time to a censored event. However, current estimators depend on the extent of follow up and many predictors using survival data lose their discriminatory power with follow up time. To account for this phenomenon we developed a concordance index based on a Pareto model. This semi-parametric model accounts for converging hazards, but leaves a baseline hazard function unspecified. In simulations under the model it was substantially less biased than other estimators. In a breast-cancer application the ordering of prognostic biomarker concordance index estimates changed when converging hazards were modelled, reflecting that some predictors are more useful for longer-term predictions than others. Our semi-parametric concordance index estimator is recommended for predictors of censored survival data when there is evidence of converging hazards.

## Appendix

### A1.1 Pareto model estimation

The Pareto model can be fitted using a semi-parametric profile-likelihood algorithm. The likelihood for an individual *i* = 1, *…*, *N* is

$$\begin{array}{c} L_{i}(\theta ,\Lambda_{0}|x_{i})=\lambda (t_{i}|x_{i};\theta {)}^{y_{i}}S(t|x_{i};\theta ) \end{array}$$

where unknown parameters are ***θ*** = (*γ*, *β*) with *γ* the Pareto parameter and *z* = *βx*, and the unknown baseline hazard function is Λ_0_. The survivor and hazard functions are given in the main paper. Then the overall log-likelihood

$$\begin{array}{c} l(\theta ,\Lambda_{0}|X_{N})=\sum_{i=1}^{n}l_{i}(\theta ,\Lambda_{0}|x_{i}) \end{array}$$

where ***X****_N_* = (*x*_1_, *…*, *x_N_*). In this semi-parametric model the baseline hazard function Λ*_i_* for *i* = 1, *…*, *N* is zero when the individual *i* is non-negative when non-censored (*y_i_* = 1) and zero elsewhere. Denote the $k=1,\ldots ,\sum_{i=1}^{N}y_{i}$ time points at which an event occurred by *s_k_*. Then differentiating the log-likelihood with respect to the unknown parameters Δ*_k_* (*y_k_* = 1) leads to a forward recursive relationship

$$\begin{array}{c} \Delta_{k+1}^{-1}=\Delta_{k}^{-1}+\sum_{i=1}^{N}I(s_{k}\leq t_{i}<s_{k+1})z_{i}(1+\gamma y_{i})(1+\gamma z_{i}\Delta_{i+}{)}^{-1} \end{array}$$

where $\Delta_{i+}=\sum_{j=1}^{i}\Delta_{j}={\hat{\Lambda }}_{0}(t_{i})$ is the estimated cumulative baseline hazard. Thus the entire baseline hazard function may be obtained based on the first point Δ_1_. The baseline hazard function may be estimated given ***θ*** by setting

$$\begin{array}{c} \frac{\text{d}l}{\text{d}\Delta_{1}}=\Delta_{1}^{-1}-\sum_{i=1}^{N}z_{i}(1+\gamma y_{i})(1+\gamma z_{i}\Delta_{i+}{)}^{-1} \end{array}$$

to zero via a root-finding algorithm. The overall profile-likelihood algorithm is to fit Λ_0_ conditional on ***θ***, and then vice versa. This method may be adjusted to account for ties; an alternative crude but effective numerical strategy is to break ties randomly.

Another approach is to use a penalised likelihood, shown to converge to the same estimate as an expectation-maximisation (EM) algorithm.^26^ It is implemented in the survival package for the statistical software R.^27^ The same approach as above may then be used to estimate the baseline hazard function.

### A1.2 Inference

Profile likelihood^20^ has been justified for inference on *β* and *γ*;^28^ the validity of a weighted bootstrap^29^ and other bootstrap weighting schemes^30^ has also been established. Bootstrap confidence intervals might also be used for the concordance index from the Pareto model. However, they will be computationally intensive because they would involve refitting the model at each resample. A less intensive approach is to obtain a valid random sample given estimation uncertainty in the parametric components $\theta^{*}=\{\text{log}(\gamma ),\beta \}$ and to compute the estimate of *C_Z_* using the random draws. Asymptotically the curvature of the profile likelihood near ***θ**** is equal to the efficient Fisher information matrix, and so one might use numerical differentiation and inversion of the profile-likelihood Hessian to obtain estimates of the covariance matrix.^20,31^ The approach is most valid when *γ* is not close to zero, and was used for the SEs given in the example. For the hybrid approach one also needs to sample from the baseline hazard function. A possible approach here is to use a piggyback bootstrap, which also samples from the parametric component, and then uses a weighted bootstrap to estimate the baseline hazard function.^32^ Alternative methods for sampling the parametric component include profile sampling using Monte-Carlo Markov-Chain simulation.^33^
